# Occurrence, Distribution, and Transmission of Alfalfa Viruses in China

**DOI:** 10.3390/v14071519

**Published:** 2022-07-12

**Authors:** Jin Li, Qiaoxia Shang, Yanqi Liu, Wenting Dai, Xin Li, Shuhua Wei, Guixin Hu, Mark Richard McNeill, Liping Ban

**Affiliations:** 1 College of Grassland Science and Technology, China Agricultural University, Beijing 100193, China; lijin2017@163.com (J.L.); bs20203240989@cau.edu.cn (Y.L.); daiwenting0513@163.com (W.D.); lixin202012@163.com (X.L.); 2 College of Bioscience and Resources Environment, Beijing University of Agriculture, Beijing 100096, China; shangqiaoxia@bac.edu.cn; 3 Institute of Plant Protection, Ningxia Academy of Agriculture and Forestry Sciences, Yinchuan 750002, China; weishuhua666@163.com; 4 Pratacultural College, Gansu Agricultural University, Lanzhou 730070, China; huguixin@gsau.edu.cn; 5 Resilient Agriculture Innovative Centre of Excellence, AgResearch, Ltd., Lincoln 7674, New Zealand; mark.mcneill@agresearch.co.nz

**Keywords:** alfalfa viruses, RNA-seq, *Odontothrips loti*, *Alfalfa mosaic virus*, *Medicago sativa* alphapartitivirus 2

## Abstract

Alfalfa (*Medicago sativa* L.) is one of the most important quality forages worldwide and is cultivated throughout China. Alfalfa is susceptible to a variety of viral diseases during its growth, which has caused huge amounts of commercial losses. However, the profile of the alfalfa virus in China remains ambiguous and the viruses transmitted by *Odontothrips loti* (Haliday), dominant insect pests in alfalfa, are also poorly understood. In the present study, virus diversity was investigated in the primary alfalfa-growing areas in China. A total of 18 alfalfa viruses were identified through RNA-sequencing (RNA-seq) and reverse transcription-polymerase chain reaction (RT-PCR). Two new plant viruses, *Medicago sativa* virus 1 (MsV1) and *Medicago sativa* luteovirus 1 (MsLV1), were detected for the first time. Another four viruses, including the Alfalfa ringspot-associated virus (ARaV), Alfalfa virus F (AVF), Alfalfa enamovirus 1 (AEV1), and Alfalfa deltaparitivirus (ADPV), were reported in China for the first time as well. Both *Alfalfa mosaic virus* (AMV) and *Medicago sativa* alphapartitivirus 2 (MsAPV2) are the dominant pathogens, with an infection incidence of 91.7–100%, and 74.4–97.2%, respectively. Additionally, *O. loti* with first- and second-instar nymphs were shown to acquire the AMV within 0.25 h of feeding on a virus-infected alfalfa. Transmission by thrips to healthy alfalfa plants was also demonstrated. Additionally, we clarified the dynamic changes in the AMV in pre-adult stages of *O. loti*, which indicated that the AMV is propagated in the nymph stage of *O. loti*. These findings provide valuable information for understanding the alfalfa virome, confirm the role thrips *O. loti* plays in alfalfa virus transmission, and improve our fundamental knowledge and management of diseases in China.

## 1. Introduction

Alfalfa (*Medicago sativa* L.) is able to be grown under a range of conditions, has high protein value, and is important in the security of livestock and poultry production systems subject to increasing climate variability [1]. It also can form a symbiosis with rhizobium and biologically fix nitrogen to provide substantial amounts of nitrogen to plants and soil and consequently reduce the need for artificial nitrogen fertilizers [1,2,3,4]. Alfalfa is an economically important forage globally, with the planting area continuing to expand, especially in China. The most recently available data show that in 2019, 4.4 million ha^2^ of alfalfa was being grown, and increased 91.3% compared to the 2.3 million ha^2^ in 2016 [5]. Containing abundant protein and micronutrients, alfalfa also becomes the natural reservoir of a large variety of viruses. To date, more than 50 viral species have been identified, with some having significant negative impacts on quality, persistence, and production, with symptoms such as dwarfism, yellowing, leaf curling, and mottling, which affect the photosynthetic efficiency [6,7,8]. Virus pathogens have been shown to have a global impact on alfalfa yield and quality [6,9]. For example, dry-weight decreased by 37–66% after being infected with the *Alfalfa mosaic virus* (AMV), followed with a 21.5% reduction in the pollen pollination rate and a 31–67% decrease in the number of nodules [10]. The primary issues we need to answer in order to control alfalfa viruses effectively are: what are the dominant viruses, which insects act as the vectors involved in transmission, and how viruses are transmitted by vectors?

High-throughput sequencing (HTS) has become undoubtedly the best way to unravel the virome of plants and animals currently [11,12,13,14]. Along with the development of HTS, both in the detection of known viruses and the discovery of new viruses, have become much more convenient [11,12,15]. So far, more than 20 viruses, including ten new species, were identified by HTS in alfalfa [6,9]. However, the studies on the virome of alfalfa comprehensively are still scarce. Up to now, only three studies of the alfalfa virome are reported, which identified four, seven, and six viruses, respectively [9,16,17]. Above all, more thorough studies are needed to better understand the viral communities in different alfalfa-growing regions to provide information for the management of alfalfa viruses.

As most plant viruses are transmitted by insect vectors, especially insects with piercing-sucking mouthparts which facilitate the efficient transmission of viruses [18,19]. Aphids, an important worldwide pest on alfalfa, is the vector for a range of viruses including AMV, *Bean leafroll virus* (BLRV), and *Alfalfa leaf curl virus* (ALCV) [20,21,22,23]. However, thrips are another major insect pest in alfalfa in China [24,25]. Thrips are equipped with rasping-sucking mouthparts, usually having high reproductive capability and a short generation time. Being small, thrips are highly locomotory and difficult to detect in the field [9,26,27]. More than ten thrips species have been documented in China, with *Odontothrips loti* (Haliday) being the most significant, with infestations resulting in a more than 20% loss in alfalfa production, as well as a 36.9% and 37.9% decrease in plant height and leaf area, respectively [25,26,28,29,30,31]. Thrips, can also transmit several plant viruses, including genera belonging to the tospovirus, ilarvirus, carmovirus, sobemovirus, and machlomovirus [32]. Transmission of the *Tomato spotted wilt virus* (TSWV) by thrips, including *Frankliniella occidentalis* (Pergande) and *Thrips tabaci* (Linderman), is the most well-known with its persistent manner by which the virus replicates and circulates throughout the various developmental stages of these thrip species [33]. While the biological characteristics and management of *O. loti* has been elucidated, the information involving its ability to transmit alfalfa viruses is poorly understood.

To investigate the occurrence and distribution of viral species present in alfalfa across China, alfalfa samples were collected from major alfalfa-growing regions, and viruses were identified by RNA-sequencing (RNA-seq), reverse transcription-polymerase chain reaction (RT-PCR), and quantitative real time PCR (qRT-PCR). The transmission efficiency of AMV by thrips was also explored to determine whether *O. loti* can successfully transmit the virus. The results obtained in this study provide valuable information for understanding the profile of the alfalfa virome and the main insects involved in virus transmission. These results contribute to the development of effective management strategies to manage viruses and their insect vectors.

## 2. Materials and Methods

### 2.1. Plant and Insect Cultures

**Figure 1:**
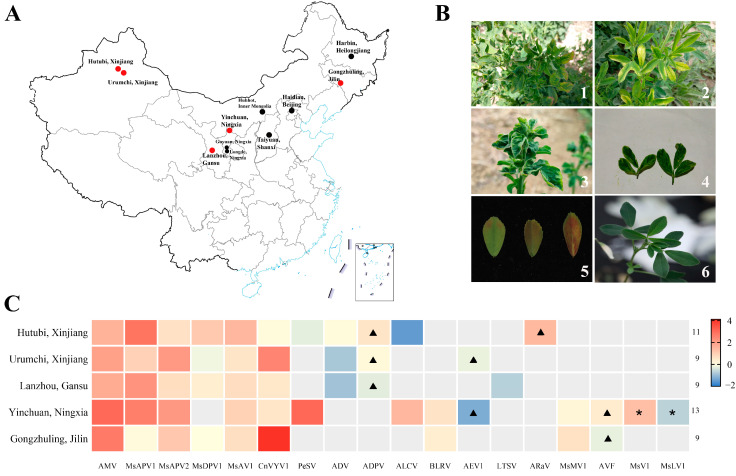
Collection of alfalfa leaves and verification of alfalfa viruses in China. (**A**) Map indicating the eight main alfalfa-growing provinces in China. The samples from the red dots were chosen for RNA-sequencing (RNA-seq). (**B**) Disease symptoms in leaves caused by alfalfa virus. 1, leaf margin reddening and vein chlorosis; 2, etiolation and mesophyll chlorosis; 3, enation; 4, striped mosaic; 5, leaf reddening; 6, asymptomatic. (**C**) Virus species and expression based on Reads Per Kilobase per Million mapped reads (RPKM) value of each region. The color is referred by RPKM value. The numbers on the right indicate the number of virus species in each region. The triangle represents the virus first reported in China. The pentagram represents a newly discovered virus.

A total of 569 leaf samples were collected from the eight main alfalfa-growing provinces (Xinjiang, Gansu, Ningxia, Inner Mongolia, Shanxi, Beijing, Jilin, and Heilongjiang) in China from 2018 to 2020. The details for sample collection are shown in Figure 1A and Table S1. From the total collection, 237 were sourced from 29 different alfalfa cultivars (Table S2), with no information on the cultivar of the remaining samples. Sampling was biased towards plants exhibiting viral disease symptoms, such as leaf margin reddening, vein chlorosis, mesophyll chlorosis, etiolation, enation, striped mosaic, and leaf reddening; however, leaves without any visible disease symptoms were also collected (Figure 1B). All samples were immersed in RNA*later*^@^ Solution (Cat. No. AM7021, Thermo Fisher, Shanghai, China) at 4 °C overnight, and then stored at −20 °C until further used.

Alfalfa *cv.* Zhongmu No. 1 used for the transmission test was cultivated in pots (15 cm high and 10 cm in diameter) containing vegetative soil and vermiculite. The pots were kept in an insect-proof environmental chamber at 26 ± 1 °C with a Light 16 h: Dark 8 h (L16:D8) photoperiod. Plants had developed through to the three true leaf stage before being used in the bioassay.

The *O. loti* colony was initially obtained from the alfalfa field at Shangzhuang Experimental Station at the China Agricultural University (E 116°11′8″; N 40°8′15″) and maintained on hyacinth bean (*Lablab purpureus* L.) pod in transparent plastic jars (10 cm high and 10 cm in diameter) with 200 mesh nylon mesh covering the jar lids in an environmental chamber at 25 °C, 70% relative humidity (RH), and a L16:D8 photoperiod. The hyacinth bean pods were sourced from a commercial supplier. Before being placed with the thrips, the pods were soaked in 0.1% NaOH solution for 1 h, followed by a triple rinse in distilled water, then air dried for 1 h.

### 2.2. Total RNA Extraction and Reverse Transcription

Alfalfa samples (100 mg) were ground in liquid nitrogen, and total RNA was extracted by using RNAprep Pure Plant Kit (Polysaccharides & Polyphenolics-rich) (Cat. No. DP441, TianGen Biotech, Beijing, China) according to standard manufacturer’s instructions. Total RNA from thrips samples was extracted using TRIzol reagent (Cat. No. 15596026, Thermo Fisher, Shanghai, China) following Manson’s protocol [34]. Extracted RNA was quantified with a Nanodrop ND-1000 spectrophotometer (Thermo Scientific, Shanghai, China). Then, the RNA (1 μg) was reverse transcribed to cDNA using PrimeScript RT reagent Kit with gDNA Eraser (Cat. No. RR047A, TaKaRa, Dalian, China) according to the manufacturer’s instructions.

### 2.3. Sequencing and Analysis Data

To understand the alfalfa virome, fifteen alfalfa leaves from five different geographical regions (Urumchi and Hutubi in Xinjiang, Lanzhou in Gansu, Yinchuan in Ningxia, Gongzhuling in Jilin, three samples per site) in autumn 2019 (Figure 1A), were processed (0.4 μg total RNA from each sample) using the rRNA-depleted RNA-Seq library construction with VAHTS mRNA-seq V3 Library Prep Kit for Illumina (Cat. No. NR611, Vazyme Biotech, Jiangsu, China), and sequenced with 150 bp paired-end sequencing on Illumina NovaSeq 6000 Sequencing System (BioMarker Technologies, Beijing, China).

The quality of reads generated was checked using FastQC (v 0.11.9). To obtain clean reads, adapters and low-quality reads were filtered out using the Sickle program (v 1.33) with the parameters “-q 30-l 50”. The clean reads were initially mapped to two reference genomes of alfalfa [35,36] using a Burrows-Wheeler Aligner (BWA) + samtools + bamtools program with default parameters [37,38,39]. The reads that were not mapped to the alfalfa genome were assembled using the SPAdes Genome Assembler software (v 3.13.0) [40]. The assembled contigs were applied as queries using the BLAST algorithm with a cut-off E-value of 1 × 10^−5^ to identify viruses. We blasted the assembled contigs against the viral database [17]. The candidate contigs were confirmed and the clean reads were mapped back the reference virus genome (see Li, et al. [17]). To understand viral expression, the Reads Per Kilobase per Million mapped reads (RPKM) is the sum of the number of reads from all samples that mapped to the sequence/number of kilobases in the sequence.

### 2.4. Phylogenetic Analysis

For the phylogenetic study, the open reading frames (ORFs) predicted by ORFfinder (https://www.ncbi.nlm.nih.gov/orffinder/, accessed on 15 January 2022) for viruses were analyzed based on the reference viral genome. Nucleotide sequences were aligned using MAGE X [41] with default parameters. A phylogenetic tree was constructed using the Maximum-Likelihood method with 1000 bootstraps by MAGE X and visualized in the Interactive Tree Of Life (iTOL) v4 [42].

### 2.5. Rearing of O. loti Nymph, Acquisition, and Transmission of AMV

To collect *O. loti* eggs, four fresh hyacinth beans pods (c. 6 cm length) were placed in plastic jars containing hundreds of mixed-sex adults. Previous methods (see Wan et al. [43]) had shown that eggs are laid on the pods. After 24 h, the pods containing the eggs were transferred to a new empty jar, which were placed in an incubator at 26 ± 1 °C with an L16:D8 photoperiod until nymphs hatched.

To study the ability of *O. loti* to acquire AMV, first- and second-instar nymphs and adults were provided with an AMV-infected alfalfa leaf in a Petri dish (14 cm-diameter) containing a moist filter paper. The insects were confined for 11 different acquisition access period (AAP): 0.25, 0.5, 1, 2, 4, 8, 12, 16, 24, 36, and 48 h, respectively. Because sourcing pure AMV-free alfalfa seeds proved to be problematic, fresh hyacinth bean pods was used as the control treatment. The AMV titers in the thrips were determined using qRT-PCR, with the treatments represented by five replicates per time period, each with five thrips.

To determine virus propagation dynamics, newly hatched nymphs, which had been provided with a 0.25 h AAP, were transferred to hyacinth beans for 0, 6, 24, 48, 96, 144, 288, 336 h, respectively. The nymphs that had not been exposed to AMV were maintained on hyacinth beans as controls. Nymphs were placed in an incubator under the condition of 26 ± 1 °C and a photoperiod of L16:D8. The AMV titers in the thrips were determined using qRT-PCR, with the treatments represented by five replicates per time period, each with five thrips.

Thrips were transferred to healthy alfalfa leaves after feeding on AMV-infected alfalfa leaves for the best AAP to understand their ability to transmit AMV. A healthy alfalfa leaf was sliced into four identical square leaves (c. 1 mm^2^), one of which was used as a control. The leaf was grouped together on a moist piece of filter paper and placed in a 5 mL centrifuge tube containing absorbent cotton moistened to minimize desiccation. The centrifuge tube was covered with 200 mesh nylon mesh to prevent the escape of thrips. After 24 h inoculation access period (IAP), thrips were removed and the leaves were incubated in the centrifuge tube for three days. qRT-PCR was used to evaluate if the leaf had been infected with AMV. There were 12 replicates per instar and five thrips per replicate.

To investigate whether there was a sex difference in the potential of adults to carry AMV, female and male thrips were collected from an alfalfa planting field. The adults were frozen and processed using the method described above. AMV titers were measured using qRT-PCR, and each sex was replicated three times with 30 adults in each replication.

### 2.6. Virus Detection

Nineteen candidate viruses were chosen for RT-PCR analysis with specific primers, which are listed in Table S3. For RT-PCR, amplifications were performed in a 25 μL reaction mixture containing 0.5 μL cDNA, 0.5 μL F/R primers (10 mM), and 12.5 μL 2 × Taq PCR Master Mix (Tiangen Biotech CO., LTD, Beijing, China). RT-PCR conditions were 3 min at 94 °C followed by 30 cycles of the 30 s at 94 °C/30 s at different annealing temperature/1 min at 72 °C, and a final extension of 5 min 72 °C. The PCR products were visualized by 1% agarose gel electrophoresis and sequenced directly in both directions by automated Sanger sequencing (Sangon Biotech, Shanghai, China). Three biological replications for every sample were independently sequenced, and assembled by SeqMan 7.1.0 (DNASTAR, Inc., Madison, WI, USA).

The incidence and prevalence of the identified main viruses based on the results of this study, in 569 samples of alfalfa leaves were investigated by qRT-PCR. For qRT-PCR analysis, a 25 mL reaction mixture, containing 0.5 μL (10 mM) of forward and reverse primers,12.5 μL of GS AntiQ qRT-PCR SYBR Green Master Mix (Cat. No. SQ412, Genesand, Beijing, China) and 1 μL cDNA, was used. The following PCR conditions were used: 5 min at 95 °C, followed by 40 cycles of 10 s at 95 °C, and 30 s at 60 °C. At the end of each qRT-PCR, the melting curve of each gene was recorded. The absolute gene expression was analyzed using the geometric mean of threshold cycle (Ct) values with the standard curve method. The protocol of construct the standard curve of each virus was showed in File S1. All primers used for qRT-PCR are presented in Table S4. The reference gene, α-tubulin (TUA) for *O. loti* and actin2 for alfalfa, were both used as normalizer for determining the relative expression of AMV when the qRT-PCR used in the transmission assay. The relative gene expression was analyzed using the geometric mean of Ct values for the reference gene with the 2^−ΔΔCt^ method [44].

### 2.7. Statistical Analysis

To calculate the incidence, the samlpe’s Ct value with none was considered as the healthy sample based on the result of qRT-PCR. The incidence of virus (%) is the number of healthy samples / the sum of samples. For each of measurement dates, the difference in viral accumulation in the leaves was examined by the Kruskal-Wallis test using nonparametric analysis of variance of the SPSS Statistics v. 22.0 (IBM, Armonk, NY, USA). The difference in the relative expression of AMV among the treatments was examined by one-way ANOVA tests using SPSS Statistics v. 22.0 (IBM, Armonk, NY, USA).

## 3. Results

### 3.1. Identification and Verification of Alfalfa Virus

Following the RNA-seq data analysis, the 64,473,364–103,607,470 clean reads were retrieved from raw reads and assembled into 3221–49,271 contigs with an average N50 length of 326 nt, after removal of alfalfa genomes (Table S5). The 19–170 contigs were blasted against 19 different plant viruses by BLAST searches (Table S6). These were AMV, *Medicago sativa* alphapartitivirus 1 (MsAPV1), *Medicago sativa* alphapartitivirus 2 (MsAPV2), *Medicago sativa* deltapartitivirus 1 (MsDPV1), *Medicago sativa* amalgavirus 1 (MsAV1), Cnidium vein yellowing virus 1 (CnVYV1), *Pea streak virus* (PeSV), *Alfalfa dwarf virus* (ADV), Alfalfa deltaparitivirus (ADPV), *Alfalfa leaf curl virus* (ALCV), *Bean leafroll virus* (BLRV), Alfalfa enamovirus 1 (AEV1), Lucerne transient streak virus (LTSV), Alfalfa ringspot-associated virus (ARaV), *Medicago sativa* marafivirus 1 (MsMV1), Alfalfa virus F (AVF), *Medicago sativa* virus 1 (MsV1), *Medicago sativa* luteovirus 1 (MsLV1), and Lettuce mosaic virus (LeMV). Following clean data mapping of the reference viruses and confirmation by RT-PCR (Figure S1), 18 viruses were identified, except LeMV (Figure 1C). Thirteen viruses were detected in Ningxia, followed in decreasing order by Xinjiang, Gansu, and Jilin provinces, respectively (Figure 1C). The top six viruses that were highly prevalent were AMV, MsAPV1, MsAPV2, MsDPV1, MsAV1, and CnVYV1. Surprisingly, both MsV1 and MsLV1 were two new viruses found in alfalfa and distributed specifically in Ningxia. Additionally, four of these 18 viruses, AVF, AEV1, ADPV, and ARaV, were identified in China.

### 3.2. Genome Analysis Viruses

#### 3.2.1. *Medicago sativa* Virus 1

**Figure 2:**
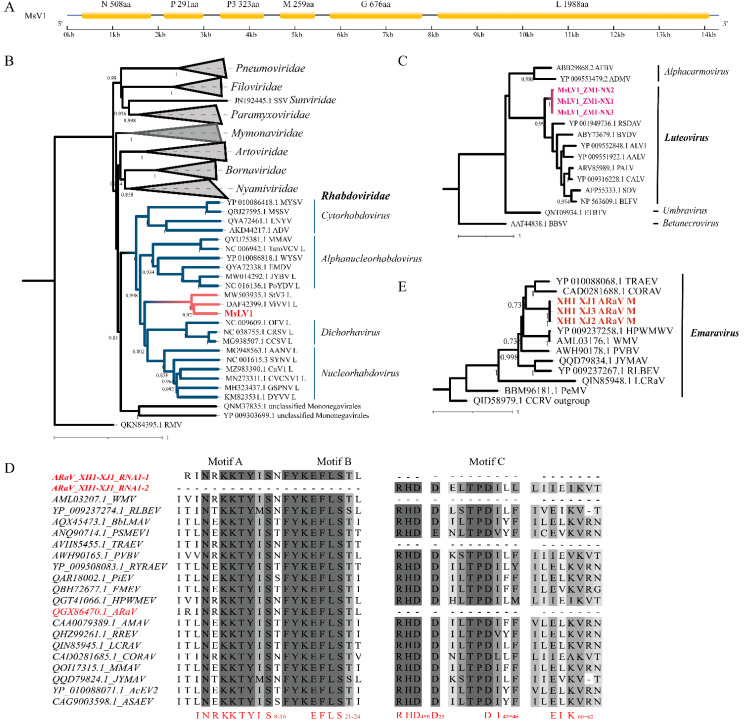
Sequence analysis of several alfalfa viruses. (**A**) Genome structures of *Medicago sativa* virus 1 (MsV1); the yellow rectangle represents the encoded ORFs, N: nucleocapsid, P: phosphoprotein, P3: hypothetical protein, M: matrix protein, G: glycoprotein, and L: the large multi-functional RNA-dependent RNA polymerase. (**B**) Phylogenic tree of the *Mononegavirales* order constructed using L. The grey branches indicated the family of the *Mononegavirales* order; the blue branch is the *Rhabdoviridae* family; the red branch is the viruses with the highest similarity to the isolate in this study; and the red label is the isolate in this study. (**C**) Phylogenetic analysis of aa sequences of *Medicago sativa* luteovirus 1 (MsLV1) RNA-dependent RNA polymerase (RdRp). The pink label is the isolate in this study. (**D**) Alignment of the conserved domain of RNA1 aa sequences in emaravirus. The red labels are the ARaV isolates, the red letters indicate conserved sites. (**E**) Phylogenetic analysis of movement protein (MP) in Alfalfa ringspot-associated virus (ARaV). The red label is the isolate in this study.

The eight and eleven assembled contigs from the Ningxia samples ZM1-NX2, 3 libraries, respectively, were most similar to *Viola verecunda* virus 1 (ViVV1, DAF42395) and Strawberry virus 3 (StV3, MW503935) belonging to the *Mononegavirales* order. All 19 contigs were assembled and predicted six ORFs including nucleocapsid (N), phosphoprotein (P), hypothetical protein (P3), matrix protein (M), glycoprotein (G), and the large multi-functional RNA-dependent RNA polymerase (L). Contigs obtained by the RNA-seq covered were not adequate to assemble into the complete genome sequence, although they contained all six ORFs matching the *Mononegavirales* order. To fill these sequence gaps, bridging primers were designed for RT-PCR based on known contigs from RNA-seq data, and the amplicons were cloned and sequenced (Table S7). The result showed that a genome of a new virus named *Medicago sativa* virus 1 (MsV1) was assembled, which comprised 14,041 nt (ON246246) (Figure 2A). These ORFs shared the 39.4–62.9% aa sequence identity with the N, P, P3, M, G, and L of StV3 and shared the 58.9–64.1% aa sequence identity with N, P3, G, and L of ViVV1 (Table S7). Meanwhile, the aa sequence similarity with other viruses of the *Mononegavirales* order was less than 40% (Table S7). The complete aa sequences of ORFs of MsV1 were phylogenetically analyzed with the members of the *Mononegavirales* order to analysis the taxonomic relationship (Figure 2B and Figure S2). The phylogenetic results revealed that the MsV1 clustered with ViVV1, StV3, and members of the *Rhabdoviridae* family based on the aa sequences of L, N, and G. The genus demarcation criteria of the *Rhabdoviridae* family is that the virus assigned to a genus form a monophyletic clade in well supported Maximum-Likelihood trees using full-length L sequences [45]. Based on the criteria, this suggests that MsV1 is a new virus belonging to the *Rhabdoviridae* family.

#### 3.2.2. *Medicago sativa* Luteovirus 1

Three assembled contigs in Ningxia samples ZM1-NX1, 2, and 3 libraries were most similar to the members of luteovirus in the *Tombusviridae* family. These contigs ranged in size between 408 nt and 812 nt and shared 59.3–66.7% aa sequence identity with the RNA-dependent RNA polymerase (RdRp) of several viruses. These belonged to the luteovirus including the Peach-associated luteovirus (PALV, ARV85989), Cherry-associated luteovirus (CALV, YP_009316228), Soybean dwarf virus (SDV, AFP55333), Bean leafroll virus (BLFV, NP_563609), Rose spring dwarf-associated virus (RSDAV, YP_001949736), and Barley yellow dwarf virus (BYDV, ABY73679) (Table S8). The aa sequences (127, 136 and 230 aa) of the partial RdRp predicted from the contigs were retrieved and phylogenetically analyzed. In Maximum-Likelihood phylograms, MsLV1 was clustered with known luteovirus, including RSDAV, BYDV, and RLBV, and separated from the alphacarmovirus, umbravirus, and betanecrovirus (Figure 2C). Multiple sequence comparison by MUSCLE alignment of luteovirus RdRP aa sequences showed that our contigs also contains the conserved sequences across the known luteovirus RdRP protein (Figure S3). Species demarcation in the luteovirus genus is based on an aa sequence difference of more than 10% for all proteins encoded [46], suggesting the *Medicago sativa* luteovirus 1 (MsLV1) as a new species.

#### 3.2.3. Other Novel Viruses

Thirty-one contigs from the Hutubi-Xinjiang samples were similar to the emaravirus belonging to the *Fimoviridae* family. Of these, 18, 10, 3, and 3 contigs were blasted from RNA1 to RNA4, respectively. The three RNA3 contigs (ON409656~ON409658) identified ARaV in our study were 1079 nt–1245 nt long sequence encoding N of 299 aa and shared the 87.3% identity of the aa sequence with the ARaV-AU-VIC329 (QGX86471). Our isolates were identified as the isolates of ARaV by the phylogenetic analysis of the nucleocapsid (N) aa sequences (Figure S4). Meanwhile, four nonoverlapping contigs assembled by 18 contigs were mapped to partial sequences of RNA1 of ARAV, encoding RdRp (MK648429). Four contigs (ON409659~ON409662) predicted 127 aa–311 aa sequences shared the 92.1–93.4% identity with the ARaV-AU-VIC329 (QGX86470) and the 30.8–61.1% aa sequence identity with the RdRp of others emaraviruses (Table S9). Multiple sequence comparison by MUSCLE alignment of the predicted protein aa sequences with the known emaravirus showed that two of four contigs contains three motifs (Figure 2D). Motif A (INRKKTYIS) and Motif B (EFLS) were both highly conserved across all emaravirus and found at aa 8–16 and 21–24 in ARaV-XH1-XJ1_RNA1-1 (ON409659). The ARaV-XH1-XJ1_RNA1-2 shared the N-terminus endonuclease domain (Motif C) with other emaraviruses and was located at RHD4-5-D25-DI45-46-EIK60-62. According to the criterion, species in the emaravirus genus are demarcated based upon the amino acid sequence of relevant gene products of RNA1 (RdRP), RNA2 (G), and RNA3 (N) differing by more than 25% [47]. Therefore, these contigs were the isolates of the ARaV in China. Three of the 31 contigs were most similar to the movement protein (MP) of the emaravirus in the *Fimoviridae* family, with a 21.4–54.1% aa sequence identity and belonged to ARaV (Table S9). These contigs (ON409653–ON409655) ranged in size among 1250 nt–1358 nt. The aa sequences of the complete MP (355 aa) were retrieved and phylogenetically analyzed (Figure 2E). In Maximum-Likelihood phylograms, those sequences clustered with the known emaravirus, most closely with Ti ringspot-associated emaravirus (TRAEV, YP_010088068) and Common oak ringspot-associated virus (CORAV, CAD0281688). In addition, two contigs blasted the RNA2 encoding G shared the 21.9–41.5% aa sequence identity with the G of several emaravirus (Table S9).

The AEV1 isolate AEV1-XN2-XJ3 (OL521668) with a length of 5659 nt encoding five ORFs shared the highest genomic identity with AEV1 (96.9%, NC_029993). ORF1 was located at 155 nt–1066 nt, encoding the putative RNA silencing suppressor (303 aa)—an ORF2 + 3 encoded fusion protein that was translated consequent to −1 programmed ribosomal frameshifting starting at 237 nt and ending at 3794 nt. This fusion protein was indicated by conserved sequence motifs encoding RdRp. ORF4 and ORF5 encoded CP (189 aa, 4008 nt–4577 nt) and the aphid transmission protein (493 aa, 4008 nt~5489 nt), respectively. The phylogenetic analysis of the deduced aa sequences of the CP genes showed that AEV1-XN2-XJ3 was an isolate of AEV1 (Figure S5A).

Two AVF isolates from Ningxia, namely AVF-ZM1-NX1 and 2, had the length of 6719 nt and 6084 nt encoding 2106 aa and 1957 aa protein sequences, respectively. Two isolates shared 78.6–78.9% genome identity and 93.4–100.0% CP identity with AVF (MG676465). According to the species demarcation in the marafivirus genus, which is based on genomic sequence differences of more than 20% and the aa of CP gene sequence difference of 10%, AVF-ZM1-NX1 and 2 were the isolates of AVF. The phylogenetic analysis of the deduced aa sequences of AVF, and the 13 other viruses belonging to the marafivirus revealed that AVF and MsMV1 were clustered together (Figure S5B). Three MsMV1 isolates, MsMV1-GN1-JL1, 2 and 3, showed 6652 nt–6701 nt encoding a 2111 aa protein from the samples of Jilin, and shared an 84.1–84.2% genomic identity with MsMV1 (MF443260). This is the first report that MsMV1 was verified by RT-PCR.

ADPV, a novel virus belonging to the deltapartitivirus genus discovered in the United States only found RNA2, was identified in seven libraries from Xinjiang and Gansu. The sequences of ADPV RNA2 with 1247 nt–1496 nt encoding the CP of 391 aa–444 aa, shared more than 99.8% identity with the reference sequence of ADPV1 RNA2 (OK514710). In addition, MsDPV1, also belonging to the deltapartitivirus genus, were identified from our samples and, therefore, we performed the phylogenetic analysis alongside with ADPV. There were ten RNA1 sequences of MsDPV1 in seven libraries from Xinjiang, Gansu and Jilin (Table S10). The sequences of MsDPV1 RNA1, which had the length of 1117 nt–1594 nt encoding the RdRp of 347 aa–477 aa (Table S10), shared more than 97.5% identity with the reference sequence of MsDPV1 RNA1 (MF443258). The seven isolates RNA2 of MsDPV1 from Xinjiang and Gansu, which had a length of 1272 nt–1469 nt encoding the CP of 401 aa, shared the more than 99.5% identity with MsDPV1 RNA2 (MF443259). The phylogenetic analysis of the RNA2 nt sequences of MsDPV1 and ADPV from our isolates and the 12 other deltapartitivirus showed that MsDPV1 was clustered with *Red clover cryptic virus* 3 (RcCV3) and ADPV was clustered with *Dactylorhiza hatagirea* deltapartitivirus (DhDPV1) (Figure S5C).

### 3.3. Incidence and Distribution of the Main Viruses

**Figure 3:**
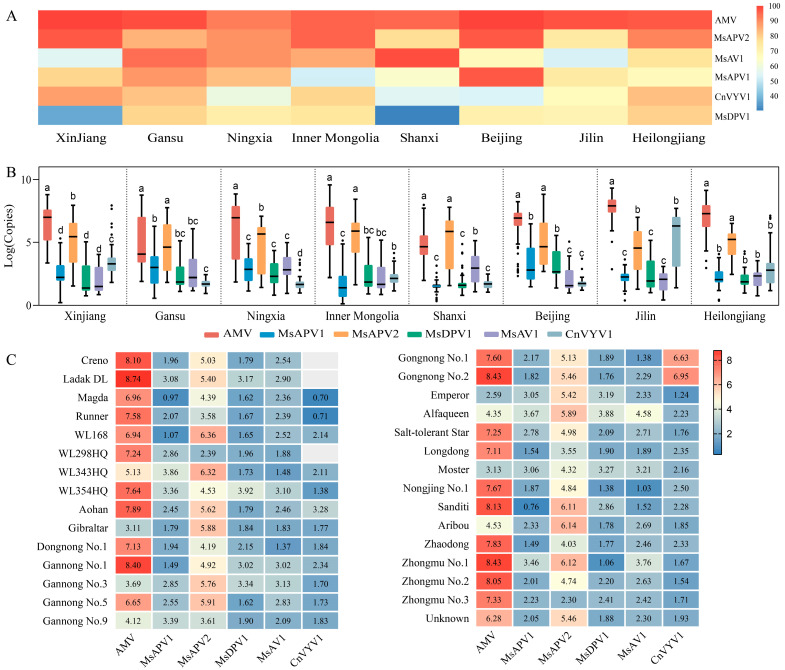
Incidence and distribution of the main six alfalfa viruses. (**A**) The incidence of alfalfa viruses in the eight alfalfa-growing provinces in China. (**B**) The distribution of viral copy numbers of alfalfa virus disease in different regions. Boxes with the same letter are not different (*p* > 0.05 in Kruskal-Wallis test) (**C**) The virus accumulation of 29 alfalfa cultivars collected across China. The value in each box is the logarithm of virus copy number. AMV: *Alfalfa mosaic virus*, MsAPV1: *Medicago sativa* alphapartitivirus 1, MsAPV2: *Medicago sativa* alphapartitivirus 2, MsDPV1: *Medicago sativa* deltapartitivirus 1, MsAV1: *Medicago sativa* amalgavirus 1, CnVYV1: Cnidium vein yellowing virus 1.

AMV, MsAPV1, MsAPV2, MsDPV1, MsAV1, and CnVYV1 were widely distributed in all eight provinces with a high infection rate among the 18 viruses identified by RNA-seq and RT-PCR. Among the six viruses detected by qRT-PCR in the 569 samples, AMV was pervasive and severe in all alfalfa-growing regions, with virus infection incidences between 91.7–100.0% (Figure 3A,B). Meanwhile, the severity of the MsAPV2 was almost as high as that of the AMV, with incidences between 74.4–97.2% (Figure 3A,B). The infection incidences of the remaining four viruses ranged from 31.1% to 98.3% (Figure 3A). In addition, it is worth noting that the CnVYV1 was more prevalent in Xinjiang and Heilongjiang than in other provinces (Figure 3A), and the CnVYV1 titers had been high in the Jilin, Heilongjiang, and Xinjiang (Figure 3B). In addition, various combinations of mixed infections were detected extensively, with a minimum of four viruses co-existing in all 569 samples (Table S11). Meanwhile, the infection profile of alfalfa cultivars showed that all 29 alfalfa cultivars were infected by AMV and MsAPV2, with significantly more viral accumulation than other four viruses (Figure 3C). Remarkably, CnVYV1 was dominantly detected in Alfalfa *cv*. Gongnong No. 1 and 2 with higher copy number of CnVYV1 compared to others 27 alfalfa cultivars.

### 3.4. Transmission of AMV by O. loti

**Figure 4:**
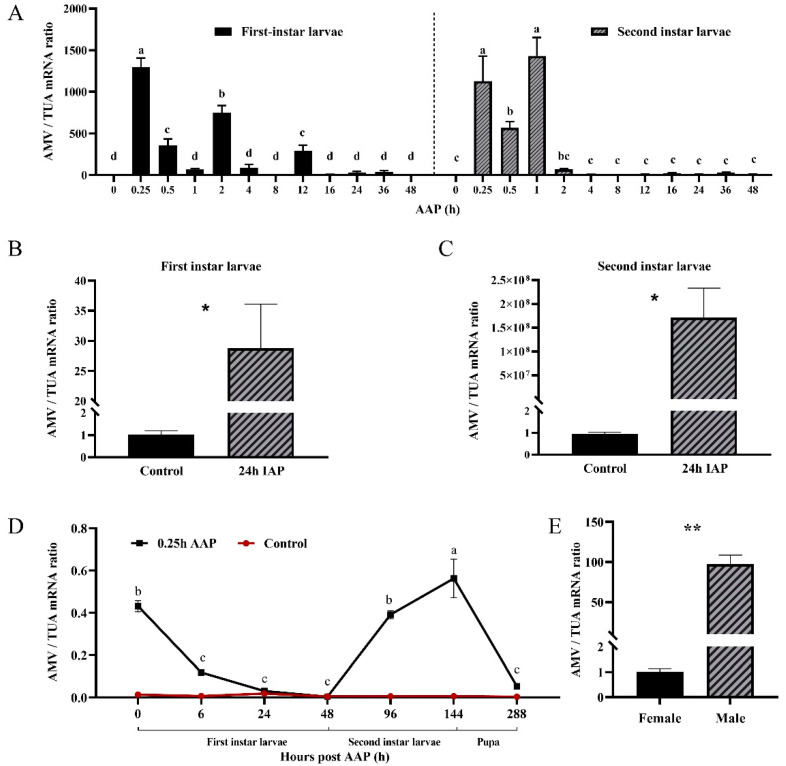
Transmission of AMV by *O. loti.* (**A**) The relative expression of AMV/TUA in the first- and second-instar nymphs of *O. loti* feeding on infected alfalfa for different acquisition access period (AAP). Transmission capacity of AMV in first-(**B**) and second-(**C**) instar nymphs of *O. loti.* (**D**) The relative expression of AMV in the pre-adult stage of *O. loti.* (**E**) The concentration of AMV found in female and male *O. loti.* Bars show mean ± SEM values and bars with the same letter are not different (*p* > 0.05 in one-way ANOVA tests); * and ** represent significant differences at *p* < 0.05 and *p* < 0.01, respectively, in one-way ANOVA tests.

The ability of *O. loti* to acquire and transmit AMV was investigated in the first- and second-instar nymphs and in adults. It was found that feeding on AMV-infected leaves for 0.25 h of AAP was enough for both the first- and second -instar nymphs of *O. loti* to acquire the AMV, with the highest titers in first-instar nymphs (Figure 4A). The maximum titer of the AMV in the second-instar nymphs was observed after 1 h of AAP, after which it dropped rapidly and stayed low for the remaining AAP (Figure 4A). Following that, transmission testing revealed that the first- and second-instar nymphs could transmit AMV after a 24 h inoculation access period (IAP) in alfalfa leaves when they reached the maximum of AMV titers. The AMV titer of alfalfa was 28 and 1.71 × 10^8^ times higher than the control, respectively (Figure 4B,C). By comparison, for adults, consistently low AMV titers were found across the different AAP by qRT-PCR (Figure S6A). Female adults were found to acquire the AMV after just 1 h AAP (Figure S6A), but failed to retain AMV after 24 h (Figure S6B). Male adults were found to acquire the AMV within 24 h AAP (Figure S6A), and could transmit the AMV into healthy alfalfa. The copy number of AMV was 20 times compared to the control (Figure S6C). The AMV expression pattern in the stage of nymphs of *O. loti* was monitored after an initial 0.25 h AAP for newly hatched nymphs on AMV-infected alfalfa (Figure 4D). The accumulation of virus in the pre-adult stages fluctuated, decreasing with time in the first instar nymphs, but then increasing in the second instar stage. The virus declined once pupation occurred. The AMV titer in male adults was found to be 97.5 times higher than female (Figure 4E).

## 4. Discussion

In our study, 18 viruses were identified and verified by RNA-seq and RT-PCR in the main planting areas of alfalfa in China. The results include two new alfalfa viruses, tentatively named MsV1 and MsLV1; four previously known viruses (AVF, AEV1, ADPV and ARaV), which we report as the first record of their presence in China, and another twelve viruses previously reported in China. This study provides a more comprehensive understanding of alfalfa virus diversity in China and updates virus species in different regions.

Meanwhile, the discovery of two new alfalfa viruses has the abundant alfalfa-virus database. One of the new viruses belonging to the *Rhabdoviridae* family, MsV1, enveloped plant and animal viruses with segmented, linear, negative-sense RNA genomes [45], with a complete genome amplified (Figure 2A). Like MsV1, the other three viruses, ADV, Medicago cytorhabdovirus A (MCVA), and Alfalfa-associated nucleorhabdovirus (AsNV), belong to cytorhabdovirus and nucleorhabdovirus genus, are known to infect plants and can be transmitted by arthropod vectors or by mechanical means [48,49]. ADV-infected alfalfa showed symptoms of shortened internodes, a bushy appearance, deformations, puckering, and vein enations. ADV was firstly identified in Argentina with a 95% disease prevalence [48]. So far, MsV1 was only detected in Ningxia, and ADV was detected in Xinjiang, Henan, Gansu, and Inner Mongolia in China [16,50,51]. Additionally, the phylogenetic result showed that MsV1 clustered with ViVV1, StV3, and these viruses might represent a new genus of the *Rhabdoviridae* family, based on the genus demarcation criteria for *Rhabdoviridae* [45]. The other new virus MsLV1 was closely similar to luteovirus in the *Tombusviridae* family (Figure 2C). The virus of luteovirus contain a single molecule of infectious, linear, positive sense ssRNA, and the genome size is fairly uniform ranging from 5.6 kb to 6.0 kb with five or six ORFs that are predicted to encode proteins [46]. However, searching the MsLV1 reads on the raw data of the Ningxia samples, in which the most MsLV1 reads were identified by mapping and iterating genome assembly, we did not find any complete genome, likely due to insufficient sequence coverage. In future research of MsLV1, the priority will be to obtain the whole genome sequence. *Bean leafroll virus* (BLRV), belonging to luteovirus, could also infect alfalfa, and be transmitted by aphids in a persistent manner [52,53]. BLRV infection in some leguminous species may cause general stunting, yellowing, and leaf rolling, with a prevalence of 25–50% in field alfalfa in China and Argentina [52,53]. Based on the above, we need to focus on isolating the MsLV1 for biological studies regarding its symptoms, transmission, and prevalence.

Four viruses, AVF, AEV1, ADPV, and ARaV, were reported in China for the first time. The first virus was ARaV showing ringspot symptoms on the leaves, which was also newly identified in Australia with partial RNA1 and RNA3 [9]. We detected partial sequences of RNA2 encoding the glycoprotein, and complete sequences of RNA4 encoding the movement protein. This was a partial supplement to the ARaV genome sequence. ARaV had only been reported in Australia and China so far [9], and its impact on alfalfa growth parameters is unknown. The second virus was AVF, belonging to the marafivirus genus, which can be transmitted by leafhoppers, beetles, and seed, and currently has only been discovered in France and China [54,55]. Like AVF, MsMV1 also belongs to the marafivirus genus, which was identified in China by HTS [54]. In this work, it was also the first report that MsMV1 has been verified by experiments [55]. The third one was AEV1, which was identified in Argentina in 2016 and showed enation and dwarfism symptoms in infected alfalfa [56,57]. In Argentina, AEV1 was detected in all 17 surveyed alfalfa-producing provinces with a prevalence of 64%, and its transmission vector was the black aphid *Aphis craccivora* (Koch) [58]. In addition, Sudan also reported AEV1 (named AEV2). AEV1 may have been introduced into China because of the importation of forage from Sudan [59]. The fourth one is ADPV, which belonged to the deltaparititivirus and was identified in alfalfa in North America.

Except for ADPV, MsDPV1 also belonged to the deltaparitivirus, which has been so far only identified in China [54]. Our results indicate that ADPV and MsDPV1 were widely prevalent in China, including Xinjiang, Gansu, Jilin, Beijing, and Hebei [17,54]. Meanwhile, MsDPV1, which was detected from the alfalfa seedling germinated for seven days in an insect-free environment, was transmitted by alfalfa seed (unpublished data),as same as the previous describes about the *Partitiviridae* family [60]. White clover cryptic virus-1 (WCCV-1), belonging to the cryspovirus (*Partitiviridae*), was transmitted vertically by the seed of *Trifolium repens*, which showed a 3–48% transmission rate in the variety cultivars and a 24% transmission rate after being stored for 50 years. Therefore, more surveillance for ADPV and MsDPV1 in alfalfa seeds is required. In addition, the ADPV sequence is incomplete, and future research may need to focus more on obtaining the complete genome sequence. For all these viruses, further studies are needed to clarify the effects on alfalfa and understand the prevalence in China.

Based on the results of RNA-seq, the top six prevalent pathogens in those alfalfa planting regions were AMV, MsAPV1, MsAPV2, MsDPV1, MsAV1, and CnVYV1, with AMV and MsAPV2 being the most widespread and serious virus-infected alfalfa in China (Figure 3A,B). In the present study, the incidence of AMV reached 91.7–100.0%, which well matched the previous field investigation of Guo et al. [16]. The incidence of AMV has significantly increased during last few years [16,61]. One reason may be that AMV is accumulated year by year in alfalfa as a perennial plant. The second reason may be that it is easily transmitted through insect vector. According to the previous study, AMV can be transmitted by 15 aphid species in a non-persistent manner, such as *Therioaphis trifolii* (Monell) and *Acyrthosiphon pisum* (Harris) in Australia [20], and *Aphis glycines* (Matsumura) in America [21]. However, according to earlier research, thrips, the major pest in Chinese alfalfa fields, could carry more AMV particles than aphids [17,27]. In this work, it was demonstrated that thrips may acquire and transmit AMV. Therefore, the annual increase in AMV accumulation might be closely relate to thrips. The incidence and the viral titer of MsAPV2, a dsRNA virus recently identified by HTS [62], has reached 74.4–97.2% (Figure 3A,B) which is drastically higher than previous data (7.8–22.1%) [16]. This finding implies that MsAPV2 is more widely distributed in China than previously reported [16]. This is most likely owing to its ability to spread widely via seeds (unpublished data). Therefore, we should pay particularly attention to the effect of MsAPV2 on alfalfa, the route of transmission, and its incidence in the field. In addition, CnVYV1, belonging to the unclassified *Picornavirales* order which contains two segments and is linear with positive sense ssRNA, was firstly isolated from *Cnidium officinale* (Makino) in Korea in 2015 [63]. CnVYV1 was recently identified by HTS from the alfalfa in Heilongjiang, located in North East China [64]. It tended to spread gradually and has a higher viral accumulation in Jilin, and in the *cv*. Gongnong No. 1 and 2, which were generally planted in the northeast of China and are cold-resistant. Therefore, the CnVYV1 may be an important virus in alfalfa crops growing in regions with low average annual temperature and is one to be monitored to reduce the virus becoming widespread.

In our study, *O. loti* was shown to transmit the AMV, with first- and second-instar nymphs being effective vectors of the virus. First- and second-instar nymphs had a strong ability to pick up the AMV, with only 0.25 h feeding on infected leaves. At the same time, the AMV could be successfully transmitted to alfalfa after 24 IAP. Especially in the second-instar nymphs, AMV titers in leaves increased by 1.71 × 10^8^ times compared to the healthy alfalfa. As the same with previous thrips-virus-transmission study, TSWV, after ingestion by first-instar nymphs, is efficiently transmitted by second-instar nymphs and adults of the thrips *F. occidentalis* [33,65]. However, the efficiency of the ingested AMV decreases with development, in that the pre-pupal and pupal stages of thrips cannot catch or transmit viruses [66,67]. In the adult stage, it was more difficult to acquire and transmit AMV than nymphs. This is consistent with the fact that *T. tabaci* and *F. occidentalis* could also acquire TSWV in the adult stage but could not transmit TSWV [67]. The reason might be that the immune system of the gut tissue barrier of the thrips adult is relatively perfect [33,66]. Remarkably, the accumulation of virus in the pre-adult stages in this data showed a downward trend at first, then upward, then lastlydownard again until the pupal stage after 0.25 h AAP of the first-instar nymphs. The same phenomenon was demonstrated in *F. occidentalis* infected by TSWV and *Impatiens Necrotic spot virus* (INSV), due to the virus titer decrease during the ecdysis stage of instar transition [68,69]. This result suggests the AMV can be propagated in the stage of nymphs of *O. loti*. Furthermore, the male adult carried much more AMV than the females, while the opposite pattern was TWSV transmission in *F. occidentalis*, in which females adults were 2–3 times more virulent than males [70]. Compared to an infected female, viruliferous male adults were on average having a longer life span, higher frequency of piercing, higher locomotory activity, and higher transmission rate [70,71,72]. In addition, a viruliferous *F. occidentalis*, which harbors a high titer of virus, is more likely to transmit TWSV multiple times [73]. Therefore, we speculate that *O. loti* males will have a higher transmission rate than females. These results would indicate that *O. loti* is an important vector for horizontal transmission of AMV in the field.

## 5. Conclusions

In conclusion, a total of 18 alfalfa viruses were verified through RNA-seq and RT-PCR. Two new plant viruses, MsV1 and MsLV1, were detected. In addition, ARaV, AVF, AEV1, and ADPV were identified and reported in China for the first time. Six main alfalfa viruses were detected in eight alfalfa planting areas by qRT-PCR. AMV and MsAPV2 are the dominant prevalent and severe pathogens, with infection incidences 91.7–100.0%, and 74.4–97.2% respectively, in the alfalfa field in China. Additionally, it is the first time that *O. loti* was proved to transmit AMV. First- and second-instar nymphs could acquire AMV within 0.25 h, and the virus can be effectively transmitted after 24 h of feeding on healthy alfalfa. We clarified the dynamic changes of AMV in pre-adult stages of *O. loti*, and suggested AMV can be propagated in the nymph stage. With respect to the field scale impacts of these viruses, these and transmission pathways need to be confirmed and quantified, and effective mitigation options developed.

## Data Availability

Sequence data are available via NCBI. The raw dataset in this study will be available in the Sequence Read Archive (SRA) repository with accession numbers SRR16477472~SRR16477486.

## Supplementary Materials

The following supporting information can be downloaded at: https://www.mdpi.com/article/10.3390/v14071519/s1, Figure S1. The coverage of reads in RNA-seq data and the confirmation by RT-PCR. (+) showed the positive result, (−) showed the negative result, whereas the red ones indicated a different with RNA-seq result. In addition, for *Alfalfa mosaic virus* (AMV), *Medicago sativa* alphapartitivirus 1 (MsAPV1), *Medicago sativa* alphapartitivirus 2 (MsAPV2), *Medicago sativa* deltapartitivirus 1 (MsDPV1) and Cnidium vein yellowing virus 1 (CnVYV1), the position of some segments was blank because they were not detected, and the segment encoding CP by RT-PCR represented the virus; Figure S2. Phylogenic tree of the Rhabdoviridae family constructed using conserved Nucleocapsid (N) (A), Phosphoprotein (P) (B), Hypothetical protein (P3) (C), Matrix protein (M) (D), Glycoprotein (G) (E). The red labels are the isolates in this study; Figure S3. The conserved sequences in MsLV1 across the RNA-dependent RNA polymerase (RdRp) protein sequences of known luteovirus. The red labels are the isolates in this study; Figure S4. Phylogenic tree of the emaravirus constructed using nucleocapsid (N) aa sequences. The red labels are the isolates in this study; Figure S5. Phylogenic tree of the viruses. (A) Alfalfa enamovirus 1 (AEV1), the red branches are all isolates of AEV1. (B) Alfalfa virus F (AVF) and *Medicago sativa* marafivirus 1 (MsMV1), the blue branches are all isolates of AVF, and the red branches are all isolates of MsMV1. (C) *Medicago sativa* deltapartitivirus 1 (MsDPV1) and Alfalfa deltaparitivirus (ADPV), the red branches are all isolates of MsDPV1 or ADPV. The red labels are the isolates in this study; Figure S6. *Odontothrips loti* acquires and transmits *Alfalfa mosaic virus* (AMV). (A) The relative expression of AMV in *O. loti* adult feeding on infected alfalfa for different acquisition access periods (AAP). Transmission capacity of AMV in female (B) and male (C) of *O. loti*; Table S1. The number of alfalfa leaves samples from eight regions; Table S2. The number of alfalfa leaf samples collected from 29 alfalfa cultivars across seven regions of China; Table S3. Primers used for RT-PCR diagnostics and sequencing validation; Table S4. Primers used for qRT-PCR diagnostics and sequencing validation; Table S5. RNA-seq summary for alfalfa samples; Table S6. The number of contigs blast viruses in the libraries; Table S7. Homology of protein sequences with viruses related to the *Mononegavirales* order; Table S8. Homology of protein sequences with viruses related to the *Tombusviridae* family; Table S9. Homology of Alfalfa ringspot-associated virus (ARaV) protein sequences with viruses related to the emaravirus; Table S10. Summary of *Medicago sativa* deltapartitivirus 1 (MsDPV1) and Alfalfa deltaparitivirus (ADPV) isolates obtained from RNA-seq; Table S11. The prevalence of multiple infections of alfalfa viruses; File S1: The protocol of constructing the standard curve.
