# Supplementary material for: Occurrence, Distribution, and Transmission of Alfalfa Viruses in China
Source: Viruses. 2022 Jul 12;14(7):1519. doi: 10.3390/v14071519 (PMC9316278; doi:10.3390/v14071519)
Supplement: Supplementary file 1 [file viruses-14-01519-s001.zip › File S1.pdf]

File S1: The protocol of constructing the standard curve.

In order to construct the standard curve, RT-PCR was performed with the viral specific primer (Table S12). For RT-PCR, amplifications were performed in a 25 μL reaction mixture containing 0.5 μL cDNA, 0.5 μL F/R primers (10 mM) and 12.5 μL 2×Taq PCR Master Mix (Tiangen Biotech CO., LTD, Beijing, China). RT-PCR conditions were 3 min at 94 °C followed by 30 cycles of 30s at 94 °C/ 30s at different annealing temperature / 1 min at 72 °C, and a final extension of 5 min 72 °C. The PCR products were analyzed by gel electrophoresis on a 1 % agarose gel/TAE gel, and sequenced directly in both directions by automated Sanger sequencing. The DNA fragment of the CP gene was amplified and cloned into a PMD18-T vector (TaKaRa, China). The positive plasmids were confirmed by sequencing. The DNA concentration of the positive plasmid was determined by Nanodrop spectrophotometer 2000 (Thermfish, USA) (Table S12) and converted to molecular copies by equation (1). The plasmids were diluted 10-fold serially to have final concentrations (Table S12). A standard DNA curve for DNA copies per reaction was generated by analyzing each dilution in triplicate by TB Green I real-time PCR. The standard curve was constructed by plotting a linear regression curve with the mean Ct values on the Y - axis and the copy number on the X-axis (Table S12).

$$\text{Molecular Copies (copies/}\mu\text{L)} = \frac{\text{DNA Concentration}\left({}^{\text{ng}}/_{\mu\text{L}}\right)\times 10^{-9}(\text{g})\times 6.023\times 10^{23}}{660\times \text{The number of bases in the plasmid}} \ldots\ldots(1)$$

Table S12. The information of specific primer, the DNA concentration, the standard curve equation and others during construction the standard curve.

Viurs Name	Specific Primer	Length of Amplifications (bp)	Length of Plasmids (bp)	DNA Concentration (ng/μL)	Initial Molecular Copies (copies/μL)	Molecular Copies of Diluted 10-fold Serially (copies/μL)	Standard Curve
AMV	F:5'-ATGAGTTCTTCACAAAAGAAAGC -3'<br>R:5'-TCAATGACGATCAAGAT -3'	666	3358	39.8	1.08×10^{10}	1.08×10^{2} - 1.08×10^{7}	y=-3.36x+40.65
MsAPV1	F:5'-TTCCCTCTAAAGCTACGGTTGT -3'<br>R:5'-CCTCATTCAGACGGGCATC -3'	822	3514	53.1	1.38×10^{10}	1.38×10^{2} - 1.38×10^{7}	y=-3.40x+34.96
MsAPV2	F:5'-TCCCTTCCAATTTCAAGTCCT -3'<br>R:5'-AGCCAAAGTGCCAGAGCC -3'	579	3271	23.8	6.64×10^{9}	6.64×10^{6} - 6.64×10^{2}	y=-3.1x+37.42
MsDPV1	F:5'-TACCGAAGCGTTCTTATCCC -3'<br>R:5'-CAGCACGATTGTCAAGCCTAAT -3'	869	3561	36.5	9.35×10^{9}	9.35×10^{1} - 9.35×10^{7}	y=-3.24x+37.84
MsAV1	F:5'-GACTACCAGAGGGAGCGGAAAC -3'<br>R:5'-CATGAGCGGCCACCAAGA -3'	430	3122	4.1	1.2×10^{9}	1.2×10^{2} - 1.2×10^{7}	y=-3.06x+36.69
CnVYV1	F:5'-GGTTATCTTGCTGGTGGTTC -3'<br>R:5'-TCCACAAAACAAGGTGCTG -3'	608	3300	43	1.19×10^{10}	1.19×10^{3} - 1.19×10^{8}	y=-3.19x+38.98
