# Supplementary figures and images for: Occurrence, Distribution, and Transmission of Alfalfa Viruses in China
Source: Viruses. 2022 Jul 12;14(7):1519. doi: 10.3390/v14071519 (PMC9316278; doi:10.3390/v14071519)

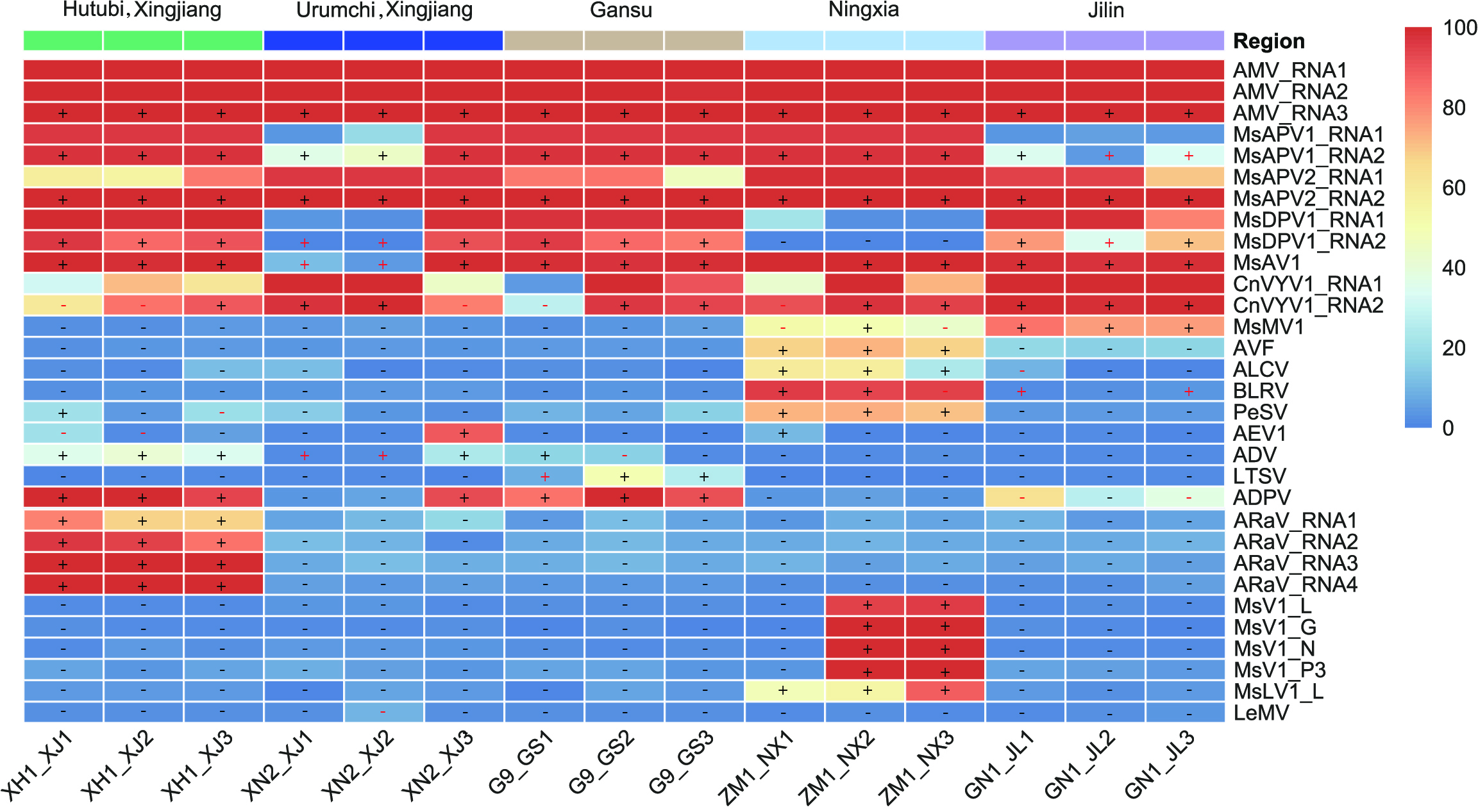

Supplement: Supplementary file 1 [file viruses-14-01519-s001.zip › Figure S1.jpg]

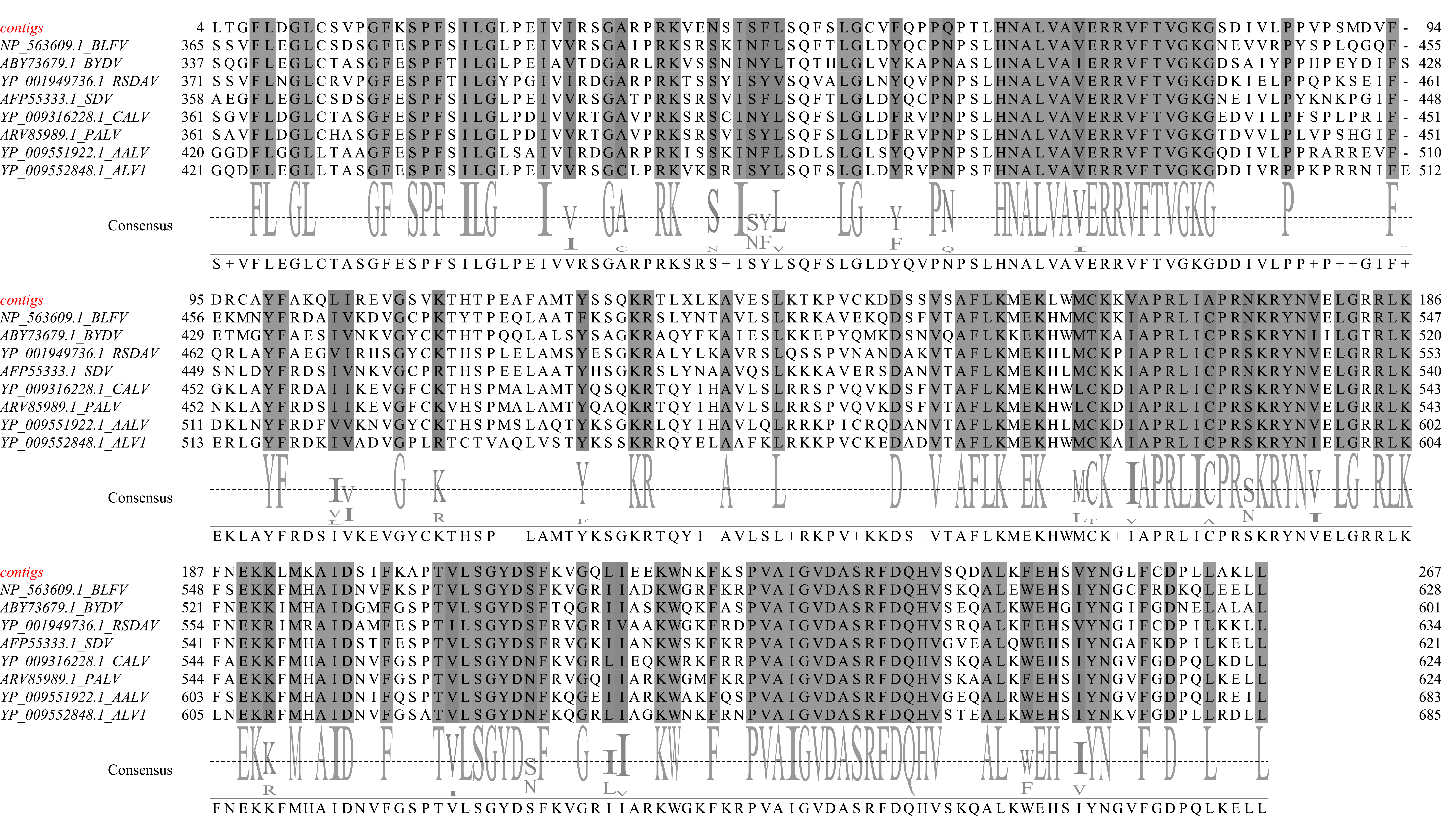

Supplement: Supplementary file 1 [file viruses-14-01519-s001.zip › Figure S3.jpg]

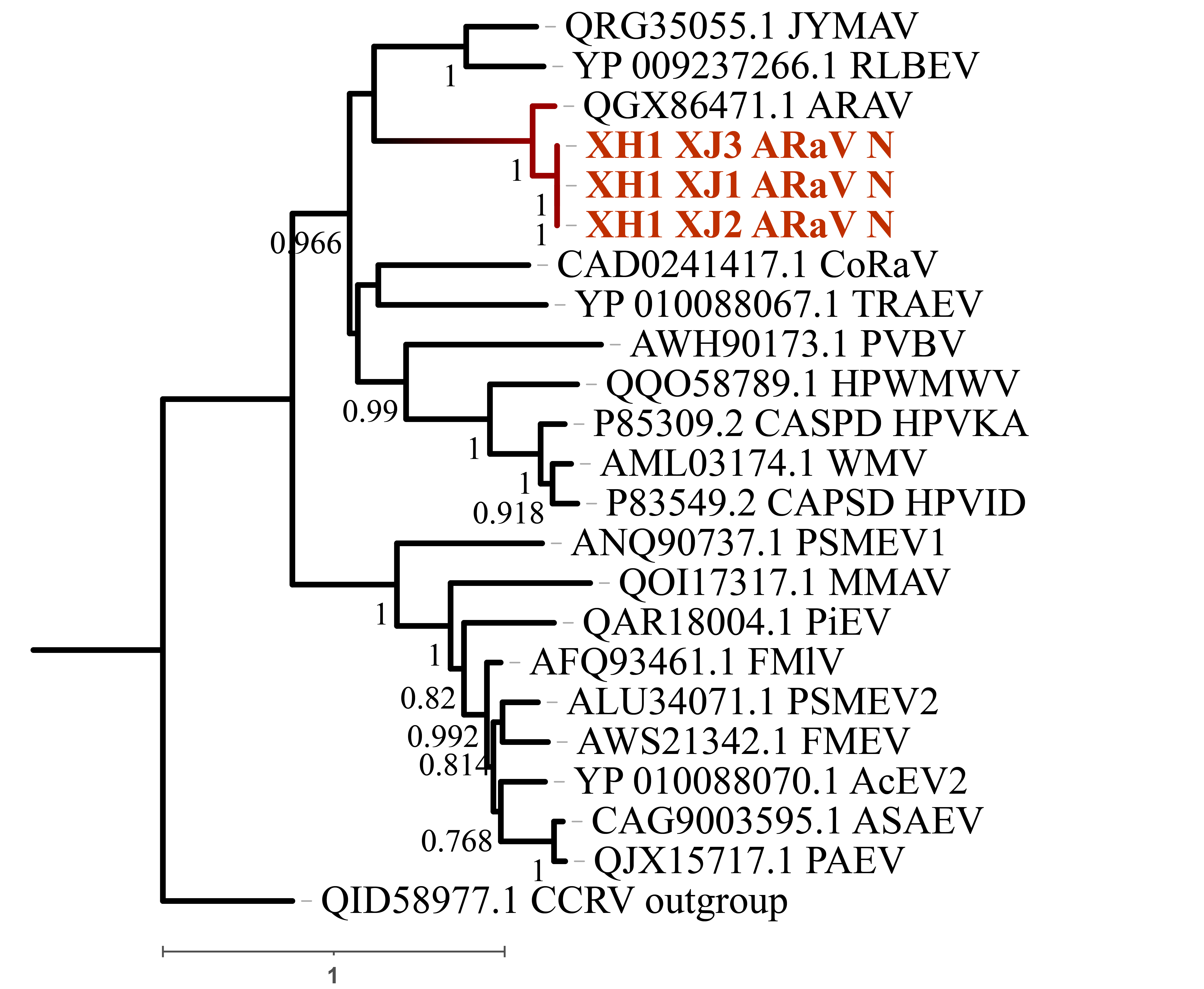

Supplement: Supplementary file 1 [file viruses-14-01519-s001.zip › Figure S4.jpg]

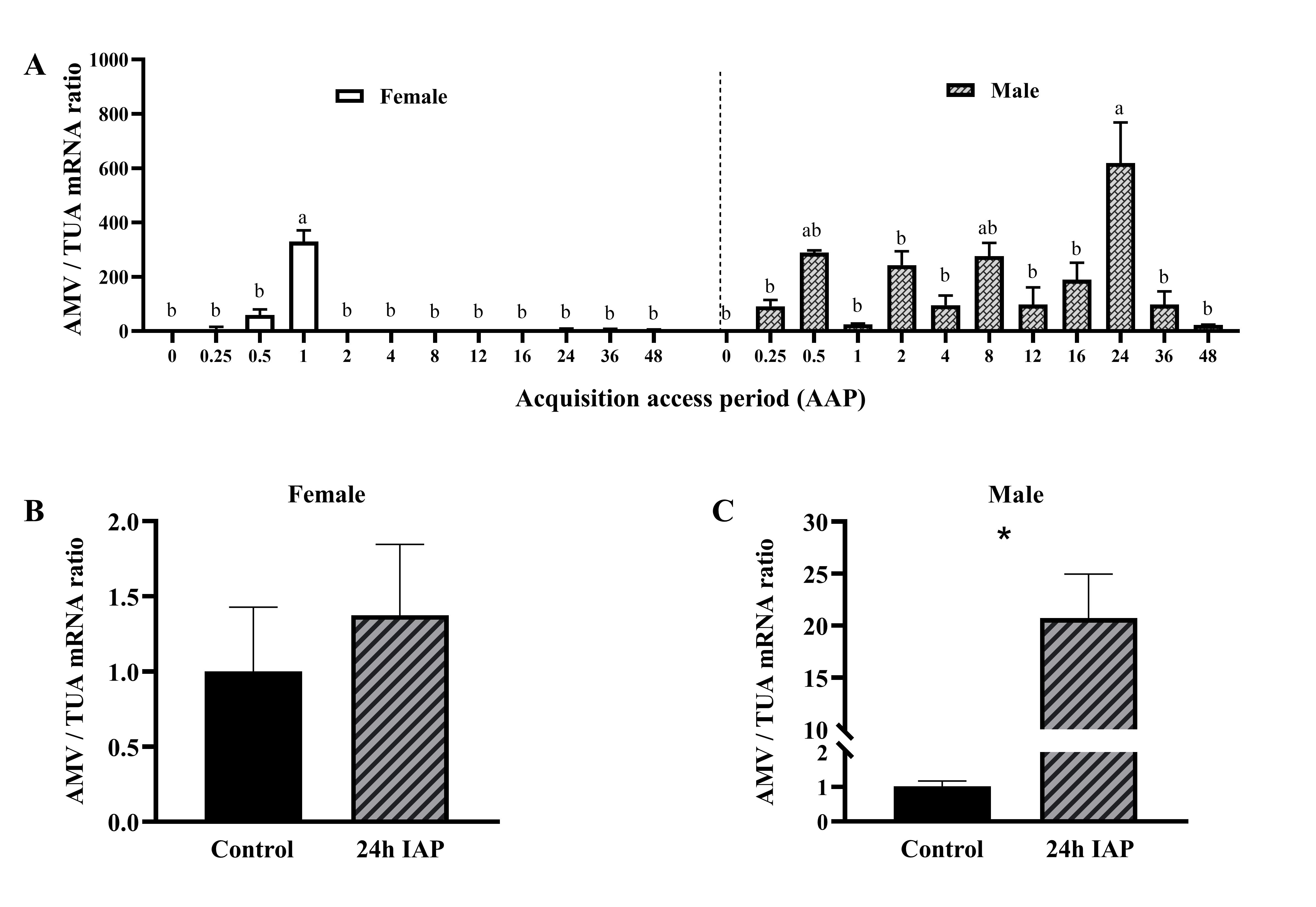

Supplement: Supplementary file 1 [file viruses-14-01519-s001.zip › Figure S6.jpg]
